# Registered nurses’ experiences of information technology use in home health care - from a sustainable development perspective

**DOI:** 10.1186/s12912-021-00583-6

**Published:** 2021-05-01

**Authors:** Vicki Koltsida, Lise-Lotte Jonasson

**Affiliations:** 1 Home health nursing, Borås municipality, Borås, Sweden; 2 Department of Nursing, School of Health and Welfare, Jönköping University, Jönköping, Sweden

## Abstract

**Background:**

The work of registered nurses in home health care is complicated and extensive, and information technology (IT) is used in everyday activities. Coordination between care and resource efficiency is important. There is a wealth of information that supports the notion of sustainable development, but what sustainable development means from the perspective of the registered nurse in home health care when using IT is limited. The term “sustainable development” is not clearly defined and is poorly researched in nursing. Sustainable development in this study includes the ecological, economic, social, technical and ethical dimensions. The aim of this study was to describe registered nurses’ experience of IT use in home health care through a sustainable development model.

**Methods:**

This study was conducted using ten semi-structured lifeworld interviews with registered nurses. The method employed was a qualitative content analysis with a deductive approach. The deductive approach consisted of a model of sustainable development.

**Results:**

Analysis of the interviews and the model of sustainable development provided categories: using IT from an ecological dimension, the registered nurses experienced reduced consumption and damage to the environment; using IT in the economical dimension, saving of time and resources was experienced; the use of IT affected social aspects such as the work environment and patient safety, and positive consequences, such as accessibility, were also mentioned; using IT from a technical dimension was characterized by the nurse’s attitude towards it – the registered nurses felt it improved the quality of care and gave users an overview of the organization; and from an ethical dimension, the registered nurses expressed the need for IT to be adaptable to the patient’s well-being and indicated that more awareness of risks in the care meeting may be needed.

**Conclusion:**

The findings are discussed based on the synergies and conflicts that arise between the different dimensions of sustainable development. IT intertwines and overlaps with, and within, the environment, economy, society, technology and ethics. Registered nurses in home health care want to conduct good and safe care, while using IT could benefit patients.

## Background

The work of registered nurses (RNs) in home health care (HHC) is complicated and extensive, and it places high demands on their skills [1, 2]. Demands can include encountering patients with both medical and nursing needs, which must be met in order for these patients to be able to live in their own homes. RNs have the responsibility to provide HHC for patients, but the work is sometimes complicated by organisational obstacles and a lack of human and financial resources, which have an impact on the work of the RN and workload [3]. The everyday activities of nurses include the use of IT (Information Technology), in assessing health for example, as well as giving treatment, conducting checkups, handling pharmaceuticals and documenting care tasks [4]. Using their skills also implies that they employ all their senses in nursing assessments [5] and are prepared to use their intuition and abilities [1, 6, 7].

Andersson and Winge [8] point out that society’s resources cannot increase at the same rate as elderly care needs in HHC. Coordination between care and resource efficiency is important and can be done, for example, with IT support and e-services, information systems, models and system components. Better home care management can support the efficient use of resources of health care institutions, producing high-quality HHC [9]. There is a wealth of information that supports the notion of sustainable development, but there is little evidence of sustainable models of care for older people and of collaboration in their application.

The model of sustainable development can be perceived as both complex and diffuse [10]. The term “sustainable development” is not clearly defined and is poorly researched in nursing [11]. But sources, such as the National Board of Health [12], report that the sustainable development model has *social, economic and ecological dimensions,* and that these determine the way we perceive the world around us. They are related to how we see the individual, the group and the community. Sustainable development also means looking at issues from a local and global perspective. The complexity of sustainable development involves not only the ecological, economic and social aspects, but also how they are integrated in practice in HHC [13–15]. Two aspects that complement the model of sustainable development have been added, i.e., *the ethical and technical dimensions* [16, 17]. This study focuses upon these five dimensions, i.e., *social, economic, ecological, ethical and technical* [13–17]. These five dimensions need to overlap and integrate in order to achieve sustainable development. Agenda 2030 emphasises that, to enable economically sustainable growth, nurses’ rights need to be protected while creating a safe and secure work environment for professionals [18]. Also, little is known about the environmental sustainability of services for older people [19]. Marimuthu and Paulose [20] describe how the emergence of sustainability in healthcare involves balancing four key factors in the operating process: environmental concerns, the needs of patients, the needs of employees and community concerns. IT contributes to sustainable development and facilitates the lives of older people through “smart” technical solutions [21].

There is a need to coordinate resources more efficiently, not only to meet the needs of today but also to deal with future challenges in elderly care. This requires integrating research results and promoting methods that increase the quality of care. This might be a solution to the problems of elderly care [22]. New requirements on improvements in HHC that support sustainable development are needed so that elderly people with complex care needs can be cared for at home [23]. Different factors can compromise the care of older people in HHC. Tingle [24] describes care in healthcare systems as a risky business, where a lack of communication, insufficient training and knowledge, as well as the health culture, can be underlying causes of inadequate patient safety. The findings of a study by Jonasson et.al [25] also revealed that technology could both be a facilitating work tool and a frustrating hindrance for RNs. Perhaps RNs’ knowledge of IT provides an understanding of how it might be changed for the good of future nursing, in the context of sustainable development [26]? The Government Offices of Sweden (2010) [27] state that IT facilitates the work environment of RNs, increases patient safety and creates opportunities for measuring organisational quality [1, 22, 27]. Today, RNs use and rely on IT daily; this would have been unthinkable only a few years ago. IT must be used in a way that facilitates their daily work, but also enables the development of HHC [27]. The nurse in HHC will conduct good and safe care if they use IT in such a way that it benefits patients, their health, and their quality of life. RNs need to learn about, and keep up with, sustainable developments. Research on nurses’ experience of IT in HHC within the context of sustainable development is limited. There is a need to develop knowledge of sustainable development to increase understanding in RNs’ work [11, 14, 26, 28]. Questions can be asked in terms of which way this can be manifested in RNs’ practice.

### Aim

The aim of this study was to describe RNs’ experience of IT use in HHC through a sustainable development model.

## Methods

### Research design and ethics

This study was conducted using a qualitative design, and the method employed was a qualitative content analysis with a deductive approach using lifeworld interviews [29]. The deductive approach consisted of a model of sustainable development consisting of five dimensions, i.e., ecological, social, economic, technical and ethical dimensions, that overlap and intertwine with and within one another [13–17].

### Sample and data collection

The study was conducted from January to March 2018 in a Swedish municipality HHC organisation, in collaboration with the regional university. HHC in Sweden is defined as the health care system that is operated within the patient’s home, which is consistent over time and planned in advance [30]. The municipal HHC system consists of RNs, physiotherapists and occupational therapists performing HHC activities [31]. The Health and Medical Service Act [32] states that health care should be offered with equivalence, respect and good quality to ensure patient security. The study took place in a city with approximately 100,000 inhabitants. The HHC organisation is divided into four geographical regions and consists of approximately 2000 patients and 200 RNs**.**

All the RNs in the municipality were informed about the study. The inclusion criteria were that the RNs had been working in a municipality HHC for at least three years and that they represented different geographical regions. The recruitment strategy was as follows: half of the informants volunteered, and half were asked to participate, always according to the inclusion criteria. The duration of the semi-structured interviews was, on average, 29 min. An attempt was made to provide as much variation as possible (Table 1).

**Table 1:** Participants’ characteristics (*N* = 10)

	n	
Participants	Total	10
	Woman	7
	Men	3
Age	Years	
	25–35	2
	36–45	4
	46–55	0
	56–65	4
Nursing experience	Years	
	1–10	3
	11–20	3
	21–30	1
	31–40	3
HHC experience	Years	
	1–5	3
	6–10	4
	11–15	1
	16–20	1
	21–25	1

Ten semi-structured lifeworld interviews were used as the data gathering method. The lifeworld theoretical perspective was chosen to understand the participants’ phenomenological experience; lifeworld information is living and concrete and, as a theoretical tool, provides an opportunity to illuminate and explain people’s ways of life [33]. An interview guide was used. The questions were: *How do you experience the use of IT in HHC? How does IT affect your daily work? Do you feel that IT development has affected HHC? What obstacles and opportunities does the use of IT present? What are your thoughts on IT use and development in terms of sustainability in HHC?* The interviews were recorded, transcribed textually and coded as informant 1, informant 2, and so on [34].

### Data analysis

Because a model existed prior to collecting data, a deductive approach [35, 36] was used for the manifest qualitative content analysis. The model became the raster (or grid) for managing the material, and the dimensions subsequently formed the categories of the study. The analysis of the interviews began with scanning the transcriptions for condensed meaning-entities through careful reading and marking of the parts that related to the purpose of the study, as the literature “demands.” This is called “open (en)coding.” Abstraction of these codes to subcategories and categories was carried out at different levels, though always in relation to the aim of the study [35, 36]. The sustainable development model and its dimensions became the theoretical framework for interpreting and managing the material, and the dimensions within the model subsequently formed the categories [13–17]. The first author, together with an assisting colleague, read all of the material several times to gain sufficient comprehension. The next step was to highlight the meaning units while preserving the core. The data were searched for specific issues or categories related to the model. The coding framework consisted of predefined categories corresponding to the questions in the interview guide (see section sample and data collection). Continuing the analysis process, the research team discussed their reflections on the codes, subcategories and categories.

## Results

### Participants

The participants were RNs and district nurses (DN). The informants (participants) consisted of seven women and three men between the ages of 28 and 64, with nursing experience of between 4 and 40 years and with 3 to 25 years of experience in HHC. The characteristics of the participants are presented in Table 1. Analysis of the interviews and the model of sustainable development provided the following categories: using IT from an ecological dimension; using IT influences the economic dimension; using IT affects social aspects; using IT from a technical dimension; and using IT from an ethical dimension.

### Using IT from an ecological dimension

A reduction of environmental damage was experienced by the RNs. The RNs found that using IT improves accessibility, in terms of communication in real time with patients, staff, other healthcare professionals and relatives. A decrease in the RNs’ need for physical travel to patients, travel times and nurses’ home visits was also mentioned, although transportation needs to be reduced further or coordinated in a more efficient way, either with a larger staffing or with other geographical placement in areas so that nurses are not too far away from the patients. A reduction of material consumption was stated. IT has reduced the use of material such as paper, which means that natural resources are being saved. The RNs believed that IT could help limit environmental damage only if harmful technical waste can be properly treated.

*“...calling a nurse and a doctor instead of taking the car saves a lot of trips...” (informant 7)*

*“... the more technical it becomes, the less paper I can say is used ...”(informant 2)*

### Using IT influences the economic dimension

The RNs experienced a saving of resources and time. IT makes work tasks more transparent, facilitates coordination and saves resources, and has made the work of HHC more effective and efficient according to the RNs. Using IT such as mobile phones/smartphones, unlocking apps, GPS systems, laptops and tablets leads to faster home visits for the RNs. IT provides and enables access to patient records, which allows for immediate access to information and documentation. The RNs felt that IT creates a more productive way of working, which facilitates the distribution of resources within the organisation and saves time. RN skills are used more efficiently and productively. Additionally, the consequences of IT use were experienced by the RNs. The cost of IT was of concern for the RNs. General medical record systems that overlap the HHC and other health care organisations and IT hardware of higher quality, which lasts longer, are preferred.

*“... IT has enabled us to help each other, we see everything that has to be done and we do it in an easier way with these schedule programmes ...” (informant 3)*

*“... medical records can be scanned; this makes it easier to not reproduce all that is said and done ...” (informant 1)*

*“... things should last longer, so we do not have to spend more money on new technology or new equipment, so ...” (informant 3)*

### Using IT technologies affects social aspects

IT has an impact on the working environment; it facilitates work, planning, safer work conditions and information flow, as experienced by the RNs. Programs that help facilitate information flow alongside other parts of the overall healthcare system were also considered significant for the work environment. The RNs wished for a faster overall development in the IT of the organisation. Patient safety issues were mentioned by the RNs. RNs experienced IT as an easy-to-use tool for the RN to practise “in the field”, offering a sense of satisfaction and security, because it improved access to information. RNs noted that patient safety can be monitored through the use of technology (through video calls, for example). Video calls became more common a few years ago, following the introduction and daily use of smartphones in HHC. Safer and more accessible collaboration with staff was also experienced by the RNs. Furthermore, the RNs mentioned consequences for availability. Responses to this item suggested that accessibility of RNs was greater, though this could have negative consequences; the RNs stated that they were sometimes interrupted in their work with patients.

*“My experience is that IT has facilitated our work... (informant 2)*

*“... using a video call is much easier for nurses to assess the patient’s situation ...” (informant 6)*

*“... IT makes us very accessible ...” (informant 3)*

### Using IT from a technical dimension

The RNs felt the approach to IT and attitude towards it were largely influenced by the users’ age, technical interest and knowledge. RNs found that it was easier to implement IT if it was seen as a tool that aided in daily nursing work. The RNs mentioned that everything will subsequently fail if IT fails, because the IT system within the nursing organisation is designed to be linked. This also affects the RNs’ work in the domestic area, because they are reliant on independent factors such as electricity, networks and technical equipment that works, otherwise the organisation becomes vulnerable. Some RNs were concerned that the IT could be used by the employer as a “monitoring instrument”, which could give a false image of the reality of the nurses’ workload. The RNs experienced an increase in health care quality. IT was considered to help with patient-centred work, streamlining and individualising patient care and, thus, improved quality of care. The RNs mentioned that IT gives an overview of the organisation and makes it easier for the nurse to obtain an overview of the workload and the way it is divided. It provides an overview of the nurses’ activities in the organisation and facilitates prioritisation of tasks.

*“... unless the IT works, the job will not work ...” (informant 10)*

*“... I think IT helps us to put the patient at the centre and improve care ...” (informant 6)*

*“.. simply to improve overview and review ...” (informant 9)*

### Using IT from an ethical dimension

When viewing IT from an ethical perspective, RNs felt that there should be an adjustment of IT to suit the patients’ well-being. Thus, IT should be adapted based on the prerogatives of nursing, where the patients are the focus and human needs are met. The RNs mentioned that IT should be an aid to nursing care and should not hinder it in any way. They felt that IT should be flexible and adaptive to individual patient needs’ and that is could be a solution to ensuring the patients’ well-being and meeting the organisation’s needs. The RNs also experienced that IT presents potential limitations and opportunities that raise ethical questions that ought to be reflected upon. An awareness of risk in the care meeting was stated: RNs said that using IT (such as video sessions/video meetings) in place of physical encounters can complicate the assessment of care and care needs. The RNs felt that the risks of IT in the caring profession need to be mentioned, because IT usage and the purpose of use need to be constantly discussed and reflected upon.

*“... I don’t like the first couple of minutes … ‘Hi, I will ...’ so you usually say that because you have to log in first... it’s the first impression that the patient gets, that you’re standing there hanging around in the hall...” (informant 3)*

*“... I still think that you should make a visit and meet face to face to assess both care needs and interventions better perhaps; it is difficult to assess through video or similar technical means...” (informant 2)*

## Discussion

The results of RNs’ experience of using IT in HHC showed that sustainable development raises discussions about conflicts and synergies between its different dimensions. The main findings indicated that IT intertwines with the environment, economy, technology, society and ethics. From a sustainable development perspective, IT can be part of the solution. Sala, Ciuffo and Nijkamp [37] point out that synergies between social, economic and environmental components need to be considered in a sustainability assessment. An overall assessment of the risks and consequences of each dimension is needed to avoid wasting opportunities for the development of future generations.

From an environmental dimension, unnecessary travel is reduced. This is confirmed by Jonasson, Holgersson, Nytomt and Josefsson [25], who believe that communication IT (such as mobile phones/smartphones) is an essential tool for the RN in HHC, providing the opportunity for counselling and teaching, as well as for getting in touch with patients, relatives, colleagues and other healthcare professionals. Other findings show that RNs see IT and communication IT as a means of increasing accessibility. Grant, Rockwood and Stennes [38] demonstrate that IT facilitates communication with patients and coordination with other colleagues in HHC, primary care and nursing staff. This might provide a partial answer to the question of how services for older people may be made environmentally sustainable [19].

From an ethical dimension, the RNs felt that IT could complicate nursing meetings when direct contact between the healthcare provider and the patient is not available. They thought that information could be lost in the assessment of caring needs when teleconferencing was used instead of physical meetings. There are advantages as well as risks involved with the use of IT for assessments in nursing meetings. This is confirmed by McCabe and Timmins [39], who state that the use of IT can lead to concerns on the part of both patients and healthcare professionals that important aspects might be overlooked.

The RNs find that IT allows for an overview that makes it possible for resources to be distributed more equitably; this will help in dealing with future challenges within elderly care. This has previously been highlighted by Josefsson and Peltonen [3], who argue for the need for more tools and improvements in HHC to ensure that RNs perform their work optimally. This could make HHC more sustainable [23]. From a technical dimension, IT increases the possibility that an overview of the organisation can be acquired, and this improves the quality of care and the wellbeing and security of RNs within a social context. A good working environment for RNs in HHC creates development potential [8].

According to our findings, RNs felt that IT facilitates contact with colleagues and other healthcare professionals, and that this increases patient safety from a social dimension. RNs working in elderly care experience stress when they feel alone in complex work situations and are frustrated by the expectation that they should know everything and should be everywhere at the same time [3]. As Jonasson, Holgersson, Nytomt and Josefsson [25] emphasise, IT and IT solutions need to be adapted to the complex work of RNs in home care. IT that is adapted to the needs of the HHC system helps ensure a safer working environment. This is consistent with the results of the present study.

There are factors that influence findings and are, therefore, a limitation of the study, such as the researcher’s background, position and perspectives [40]. The study used a deductive approach, which has its difficulties, because the concept of sustainable development can be perceived as both complex and diffuse, and the authors feel that this presents another limitation for the study. To overcome this, dialogue and reflection were used in the research group and in a seminar with other researchers. Other limitations of the study could be the sample size and the time of IT usage within the organisation. The main strength of this study is that the RNs interviewed came from different workplaces in the municipality and in the HHC organisations, so the data sources were varied. Kvale and Brinkman [41] have indicated that the quality of interviews is improved if the interviewer has good subject knowledge, the interview is structured and participants are aware of the purpose of the interview. Guidelines compensate for a lack of experience in interviewing technique, which may also be considered a limitation. Validity can be reinforced by variations in the participants’ age, sex and professional experience, and also in the sample size of ten participants. An interview guide with semi-structured questions was used to keep the focus on the issues at hand. According to Sandelowski and Leeman [42], the transferability of the results can be handed over to the person who reads, because it is the reader who determines the transferability to another context.

## Conclusions

Sustainable development and the understanding of what that model means for RNs in HHC when using IT is limited among researchers. This study discusses the use of IT from an ecological, economic, social, technical and ethical dimension. According to RNs in HHC, there are indications that the use of IT can decrease consumption and damage to the environment, and benefits users through the saving of time and resources. Furthermore, the work environment and patient safety can be developed by IT. The attitudes of RNs towards the use of IT vary. From an ethical dimension, IT needs to be adapted to the patient’s well-being, and more awareness may be needed of ethical assessment in nursing meetings. RNs in HHC want to conduct good and safe care, whilst using IT could benefit patients.

## Data Availability

For ethical reasons, the raw data cannot be shared.

## Abbreviations

IT: Information Technology
HHC: Home Health Care
RN: Registered Nurse
